# C-reactive protein levels and prevalence of leukopenia in patients with inflammatory bowel disease treated with azathioprine and/or mesalazine: a real-life study

**DOI:** 10.31744/einstein_journal/2022AO6500

**Published:** 2022-04-25

**Authors:** Rejani Cristine Faustino dos Santos, Wilson Roberto Catapani, André Akira Ramos Takahashi, Jaques Waisberg

**Affiliations:** 1 Hospital Universitário Universidade Federal da Grande Dourados Dourados MS Brazil Hospital Universitário, Universidade Federal da Grande Dourados, Dourados, MS, Brazil.; 2 Centro Universitário FMABC Santo André SP Brazil Centro Universitário FMABC, Santo André, SP, Brazil.

**Keywords:** Crohn disease, Proctocolitis, Azathioprine, Mesalamine, Leukopenia, C-reactive protein, Colitis, ulcerative, Inflammatory bowel diseases

## Abstract

**Objective:**

To examine serum C-reactive protein levels and the prevalence of leukopenia in patients with Crohn’s disease or ulcerative colitis undergoing treatment with azathioprine and/or mesalazine.

**Methods:**

Retrospective observational study based on clinical and laboratory data collected from medical records of 76 adult patients with inflammatory bowel disease treated with azathioprine, mesalazine or both. Sex, age, diagnosis, number of blood samples and elevated serum C-reactive protein levels during the follow-up period were recorded. The following variables were analyzed in terms of C-reactive protein levels and leukopenia episodes: sex, age, diagnosis of inflammatory bowel disease and type of drug. Statistical analyses included multiple logistic regression and the Fisher’s exact test for qualitative variables.

**Results:**

Leukopenia was observed in 18.4% of patients and was associated with older age and higher doses of medication. In 44% of patients, C-reactive protein levels were high. However, symptoms were not associated with abnormal levels of this marker.

**Conclusion:**

Regardless of symptoms, serum C-reactive protein levels were not a reliable indicator of controlled inflammatory bowel disease. Leukopenia was independently associated with older age and higher doses of medication and is a common side effect, which should be routinely monitored.

## INTRODUCTION

Crohn’s disease and ulcerative colitis are chronic incurable diseases characterized by inflammation of different intestinal segments, which require long-term treatment.^(1)^ For successful treatment, therapy must be individualized according to the level of disease activity, affected bowel segment and disease extent, in an effort to alter the course of the disease, resolve the inflammation and minimize the adverse effects of therapy.^(2,3)^ Treatment goals in patients with inflammatory bowel disease (IBD) have evolved from simple control of disease symptoms, which is no longer a satisfactory therapeutic goal, to normalization of inflammatory laboratory test parameters (biochemical or laboratory remission) and intestinal mucosal healing, or deep remission.^(4)^ Colombel et al. reported enhanced disease control when therapy with adalimumab was adjusted according to C-reactive protein (CRP, a marker of inflammatory activity) levels rather than symptoms.^(5)^

Mesalazine (MSZ) and azathioprine (AZA) are some of the drugs that can be used to treat IBD. Mesalazine plays an important role in the treatment of ulcerative colitis and is the drug of choice in many cases. Azathioprine can be used in mild to moderate cases of Crohn’s disease and in cases of ulcerative colitis refractory to MSZ.^(2,3)^ However, complications associated with these drugs, such as leukopenia and pancreatitis, have been known for a long time.^(5,6)^In order to ensure treatment safety and efficacy, it is imperative to understand their toxic potential and to properly monitor patients. Brazilian studies investigating the ability of these drugs to control inflammation in IBD are scarce.^(7,8)^

The main hypothesis in this study is that patients with Crohn’s disease or ulcerative colitis treated with MSZ and AZA in public outpatient services do not achieve satisfactory outcomes regarding disease remission, beyond symptomatic and biochemical control.

## OBJECTIVE

To examine serum C-reactive protein levels and the prevalence of leukopenia in outpatients with Crohn’s disease or ulcerative colitis undergoing treatment with azathioprine and/or mesalazine.

## METHODS

A retrospective, observational longitudinal study based on clinical and laboratory data of 76 adult male and female patients treated with MSZ or AZA (ulcerative colitis patients) or AZA (Crohn’s patients). Patient data were extracted from medical records.

The assessment period was defined as the time (days) elapsed from the first to the last laboratory tests performed in each case.

The following inclusion criteria were adopted: diagnosis of Crohn’s disease or ulcerative colitis confirmed by clinical, radiological, laboratory and endoscopic findings, age over 18 years, treatment with a stable dose of MSZ or AZA throughout the experimental period, minimum follow-up of 4 months and at least two blood samples collected during the experimental period. Exclusion criteria were as follows: concurrent use of any other drug to treat IBD, fulminant IBD, infection and pregnancy.

Leukopenia was defined as leukocyte counts <4,000 cells/mL. The expected plasmatic CRP level was ≤8mg/dL, according to the reference value adopted by the laboratory. For statistical analysis purposes, patients were allocated to one of two groups: patients with normal blood test results (Normal Group) and patients with at least one abnormal laboratory test result (abnormal CRP level or leukopenia) over the course of the assessment period (Abnormal Group).

Statistical analyses were carried out using Graph Pad Prism 8 (Graph Pad Prism, San Diego, CA, USA). Independent associations between variables and outcomes (*i.e*., prevalence of leukopenia and abnormal CRP levels) were investigated using multiple logistic regression. Variables were chosen as follows: diagnosis, age (years), sex, duration of follow-up (days) and drugs used (AZA, MSZ or both).

This study was performed at the Department of Gastroenterology, *Faculdade de Medicina do ABC* (Santo André, São Paulo, Brazil) and was approved by the institutional Research Ethics Committee #3.315.832, CAAE: 09029219.0.0000.0082.

## RESULTS

Female patients (53.4%) prevailed in this sample of 76 patients. Twenty-six patients with Crohn’s disease, 15 (57.6%) were females and 11 (42.4 %) were males. Fifty patients with ulcerative colitis, 24 (48%) were females and 26 (52%) were males. All patients in this sample were Caucasian.

Patients with Crohn’s disease were aged 49.9±14.6 years (20-73 years). Patients with ulcerative colitis were aged 48.2±16.0 years (19 to 86 years).

The number of blood samples collected per patient and plasma CRP level measurement ranged from 2-9 (mean, 4). Follow-up assessments were scheduled at intervals ranging from 141-678 days (mean, 423 days). The dose of AZA ranged from 100-350mg (20-3.7mg/kg) per day per patient. The dose of MSZ dose ranged from 1.0-3.6g per day per patient.

The number of episodes of leukopenia ranged from 0-4 per patient. Leukopenia was detected in at least one blood sample test in 14 out of 76 (18.4%) patients.

Leukopenia was independently associated with age and daily dose of medication (Table 1). Of patients treated with 2g/day of MSZ, seven were also taking 100-150mg/day of AZA. Of patients treated with 2.0-4.0g/day of MSZ, five were also taking 150-300mg/day of AZA.

**Table 1:** Multiple logistic regression. Associations between episodes of leukopenia and independent variables

Variable	95%CI	Odds ratio	Z	p value
Age	0.95-1.05	1.00	2.40	0.016
Sex	0.71-14.26	2.94	0.16	0.868
Diagnosis	0.05-1.18	0.27	1.44	0.149
Number of samples	0.60-1.42	0.94	1.68	0.091
Assessment period	1.00-1.01	1.00	0.28	0.772
Daily AZA dose	1.00-1.02	1.01	2.32	0.020
Daily MSZ dose	0,63-1.87	1.09	2.13	0.033

95%CI: 95% confidence interval; Z: estimated standard error; AZA: azathioprine; MSZ: mesalazine.

Sex was the only variable associated with abnormal CRP test results during the assessment period (Table 2). Approximately 44% of patients had at least one abnormal CRP test result during the assessment period.

**Table 2:** Multiple logistic regression. Associations between abnormal C-reactive protein levels and independent variables

Variable	Odds ratio	95%CI	p value
Age	1.018	0.9855-1.052	0.2917
Sex	3.330	1.175-10.10	0.0272
Diagnosis	0.9427	0.3066-2.879	0.9169
Number of samples	1.408	0.9895-2.074	0.0658
Assessment period	1.001	0.9973-1.005	0.5134
Daily AZA dose	0.9998	0.9925-1.007	0.9482
Daily MSZ dose	1.160	0.7676-1.756	0.4773

95%CI: confidence interval; AZA: azathioprine; MSZ: mesalazine.

Over the course of the assessment period, 31 patients remained asymptomatic, whereas 45 had abdominal pain, diarrhea or bloody stools.

Of the 31 asymptomatic patients, 12 had at least one abnormal CRP test results. In contrast, of the 45 symptomatic patients, 20 had at least one abnormal CRP test results (Table 3).

**Table 3:** Relation between symptoms and abnormal C-reactive protein test results over the course of the assessment period

Symptoms	At least one abnormal CRP n (%)	No abnormal CRP n (%)	p value
Yes	20 (44.4)	25 (55.6)	0.64
No	12 (38.7)	19 (61.3)	

Fisher´s exact test; CRP: C-reactive protein.

## DISCUSSION

Inflammatory bowel disease diagnosis is based on symptoms, physical examination and ancillary procedures, such as colonoscopy, abdominal magnetic resonance imaging and computed tomography.^(1,2)^ Colonoscopy is the gold standard for investigation of ulcerative colitis, since lesions are limited to the colon and rectum. Approximately 70 to 80% of Crohn’s disease lesions are found in the terminal ileum or colon and can be seen on endoscopic examination.^(2,3)^ Ulcerative colitis and Crohn’s disease share several histologic features. Therefore, hystologic findings are not pathognomonic or either condition^(3,4)^ and multiple biopsy specimens must be collected from the ileum, colon, and rectum for greater diagnostic accuracy. As shall be discussed later, markers of inflammation such as CRP and fecal calprotectin are also nonspecific and are mostly used to determine treatment effectiveness, in an effort to avoid repeated colonoscopies.^(1,3)^

Real-world studies investigating IBD treatment in Brazil are scarce and there is an unmet need for related data. Colonoscopy is the gold standard for mucosal inflammation assessment. However, colonoscopy is not widely available, particularly in public health care settings. Therefore, a putative marker of inflammation CRP was used in this retrospective study. Moreover, patients with IBD often suffer from lactose intolerance and/or irritable bowel syndrome, which can also manifest as diarrhea and abdominal pain. For these reasons, symptoms are not good indicators of disease activity. The use of CRP to investigate IBD is common practice.^(5)^

The number of blood samples collected during the assessment period varied between patients in this sample. This may have introduced a bias in the analysis regarding frequency of abnormal CRP test results and platelet counts. Hence, patients were allocated to different groups according to laboratory test results (normal CRP levels and platelet counts or at least one abnormal test result). Abnormal test results were not significantly associated with the number of samples collected.

Efforts were made to detect trends in CRP levels during a specific period. In this sample, 38.7% of asymptomatic patients had at least one abnormal CRP test result during the assessment period. In contrast, 55.6% of symptomatic patients did not. Ideally, the intestinal mucosa should be examined using colonoscopy. However, as previously alluded to, this study was based on data collected in public health system settings, where colonoscopy is not widely available. For this reason, a putative marker of inflammation (in this case, CRP) was used instead of endoscopic examination. C-reactive protein levels may rise in response to several factors. Still, CRP is thought to be a useful marker of IBD.^(5)^

Leukopenia is a major long-term adverse effect of treatment with AZA and/or MSZ.^(5,6,9)^ Colli et al.^(10)^ reported leukopenia in 36 out of 106 (34%) patients with IBD treated with AZA, with a mean time to onset of leukopenia of 35 months after initiation of treatment. Lémann et al.^(11)^ evaluated 87 patients with long-term IBD and found a prevalence of leukopenia of 24.5%. Constantino et al.^(12)^ also detected leukopenia in 12 out of 266 (4.5%) patients with IBD. In this study, leukopenia events were associated with increasing age and daily dose of medication, but not with sex or diagnosis. The overall prevalence of leukopenia was 18.4%. Indeed, different factors may impact leukopenia development, including different phenotypes of AZT-metabolizing enzymes, which are genetically determined and vary according to ethnicity.^(13)^

A Brazilian study carried out by Chebli et al.^(7)^ revealed that AZA was able to sustain clinical remission (*i.e*., not confirmed by endoscopic examination) in 55%, 52%, and 45% of patients with ulcerative colitis (first, second and third year of treatment respectively).^(7)^ Pinto et al.^(8)^ reported that, in cases of Crohn’s disease, the proportion of corticosteroid-dependent patients achieving clinical remission with AZA treatment at the 1-year follow-up was 61%. Of note, Chebli et al.^(7)^ and Pinto et al.^(8)^ used clinical parameters to distinguish between remission and disease activity, whereas a laboratory marker (CRP level) was used in this study. Therefore, studies cannot be directly compared.

A critical factor that should be accounted for is treatment adherence. Different from clinical studies, in which medication use is monitored, non-adherence to treatment is relatively common in real life (up to 45%)^(14)^ and is associated with treatment failure.

This study has some limitations. Sample size was relatively small due to incomplete medical records (*i.e*., lack of blood cell counts and CRP level measurements in sufficient numbers) and limited number of patients using stable doses of medication. Many patients attending scheduled follow-up appointments failed to present blood test results. Also, the fact that treatment adherence could not be evaluated may have significantly impacted results, since noncompliance is quite common in long-term treatment of any disease. Other reasons for noncompliance are difficulties and delays to obtain medications supplied by the public healthcare system and poor compliance with prescription recommendations due to lack of understanding. In this study, CRP test results were assumed to reflect inflammation associated with the underlying disease, since no other potential causes of abnormal tests results were found in medical records.

In summary, this study revealed that many patients seen in public healthcare system settings fail to maintain normal CRP levels, which are thought to be a putative marker of inflammation, regardless of symptoms. Apparently, in a sizeable proportion of these patients mucosal healing was not achieved for some reason. Noncompliance with treatment may explain this finding. Leukopenia occurred in 18.4% of patients and was independently associated with increasing age and higher doses of medication. This is a common side effect, which should be routinely monitored.

## CONCLUSION

Regardless of symptoms, serum levels of C-reactive protein were not a useful marker of controlled inflammatory bowel disease. Leukopenia was independently associated with increasing age and higher doses of medication and is a common side effect, which should be routinely monitored.
